# Supplementary material for: Proposal for nanoscale cascaded plasmonic majority gates for non-Boolean computation
Source: Sci Rep. 2017 Dec 19;7:17866. doi: 10.1038/s41598-017-17954-2 (PMC5736723; doi:10.1038/s41598-017-17954-2)
Supplement: Supplementary file 1 — Supplementary Information [file 41598_2017_17954_MOESM1_ESM.pdf]

Proposal for nanoscale cascaded plasmonic majority gates for non-boolean computation – Supplementary Information

Sourav Dutta[1*], Odysseas Zografos[2], Surya Gurunarayanan[2], Iuliana Radu[2], Bart Soree[2], Francky Catthoor[2], Azad Naeemi[1]

[1]School of Electrical and Computer Engineering, Georgia Institute of Technology, Atlanta, Georgia 30332, USA
[2]IMEC, Leuven, Belgium

*Correspondence to sdutta38@gatech.edu

S1. Excitation of SPP

On-chip propagating SPPs can be launched in several ways - optically by focusing external laser radiation on grating couplers [1-3] or electrically using light-emitting diodes [4,5], Si-based electrical source [6] and electron tunneling [7-9]. Phase coherency is an important requirement for our 3-input majority logic operation. Currently, phase coherent waves are not yet demonstrated for two independent emitters. However, provided sufficient input power, by splitting the output of a single emitter into three components and injecting them into the input waveguides, phase coherency can be assured. Since we rely on the phase of the SPP wave as the state or computational variable, the information can be written into the phase of wave in each of the three individual waveguides of the majority logic by incorporating phase modulators such as Mach-Zehnder interferometers (MZI) [10] or shift-keying based resonators [11]. However, since the emphasis of this work is on building a nanoscale cascadable plasmonic majority logic, we do not refer to a particular technique of excitation and use the generalized mode source in Lumerical Solutions[12] to inject a fundamental guided mode into the plasmonic waveguide with the phase of the source specified as either 0 (ϕ) or 180° ($\phi + \pi$) for simulating a logic 1 or 0, respectively.

S2. Characterizing mode source

Surface plasmon polaritons were excited using the standard mode source in Lumerical Solutions[12] to inject a fundamental guided mode into the plasmonic waveguide. The standard mode source injects a broadband Gaussian pulse signal as shown in Fig. S1(a). Fig. S1(b, c) shows the spectrum of the excitation signal with a center wavelength of 1550 nm and center frequency of 193 THz with a bandwidth of 43 THz. We use this excitation for our simulation throughout the manuscript unless specified.

We additionally ran simulations changing the mode source from a broadband to a narrowband by changing the pulse duration of the Gaussian pulse signal as shown in Fig. S1(d). Fig. S1(e, f) shows the spectrum of the excitation signal with a center wavelength of 1550 nm and center frequency of 193 THz with a bandwidth of 10.7 THz. The simulation results for a 3-input majority

logic gate using this narrowband excitation signal is shown in supplementary section S8. The results and the conclusion of the paper remain same for either of the mode sources.

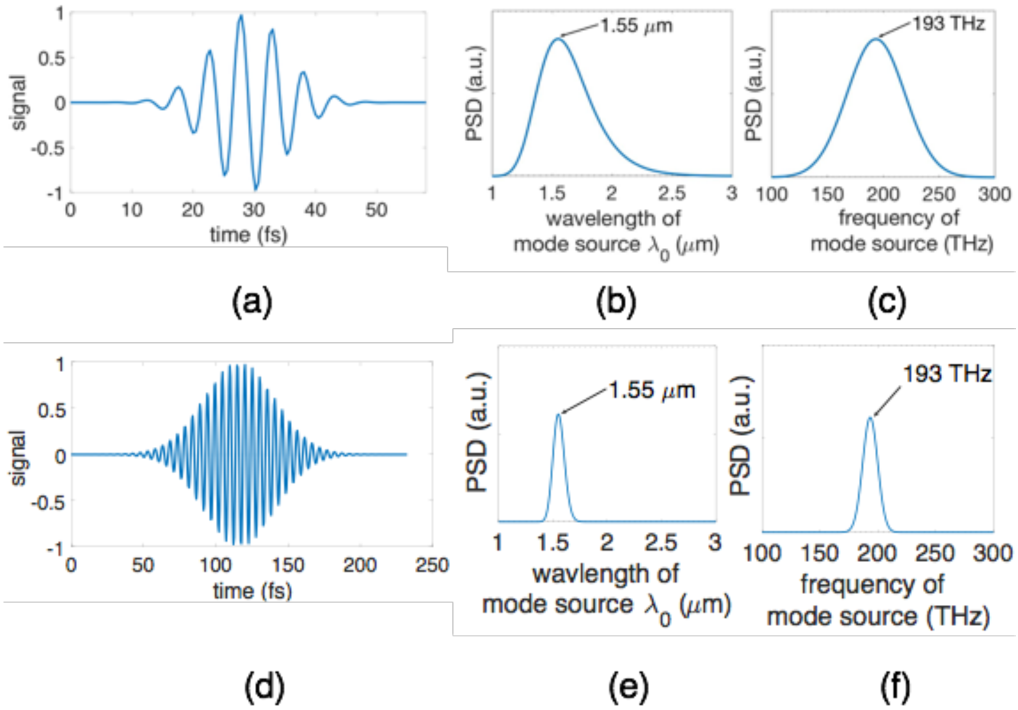

Figure S1. (a) Broadband Gaussian pulse signal injected by the standard mode source in Lumerical simulation. (b, c) Spectrum of the excitation signal with a center wavelength of 1550 nm and center frequency of 193 THz with a bandwidth of 43 THz. (d) Narrowband Gaussian pulse signal injected by the standard mode source in Lumerical simulation. (b, c) Spectrum of the excitation signal with a center wavelength of 1550 nm and center frequency of 193 THz with a bandwidth of 10.7 THz.

S3. Impact of gap width of the output waveguide on the transmitted power

Fig. S2(a) shows the increase in the transmitted output power (normalized to the source) with the increase in the gap width of the output waveguide. This results in a considerable reduction of the backflow of power occurring due to reflection from the merging point. The increase in transmission due to wider output gap width can be further explained by resorting to an approach of impedance matching put forward by Cai *et. al* [13]. At wavelengths considerably larger than the size of the structure where the absorption is negligible (above the visible spectrum for silver), one can use the quasi-static approximation treating the waveguides as equivalent transmission lines with some characteristic impedance [13-15]. Following the approach highlighted in ref. [13] for 3-D waveguides, we calculate the direct integrals for the transverse electromagnetic fields to evaluate the effective voltage $v = \int_{-\infty}^{+\infty} E_y d_y$ and current $I = \int_{-\infty}^{+\infty} H_z d_z$ and hence the equivalent impedance $Z = \frac{v}{I}$. Fig. S2(b) illustrates the reason for the increase in the transmission

due to an increase in the impedance of the waveguide (calculated from Lumerical simulations) with the output gap width.

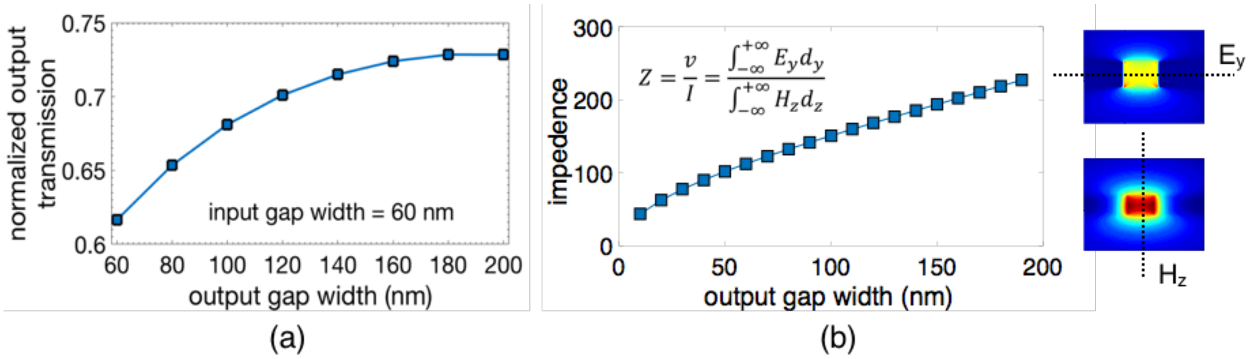

Figure S2. (a) Simulation result showing the increase in the normalized output transmitted power with the increase in the gap width of the output waveguide. (b) Calculated impedance of the waveguide as function of the gap width.

S4. Time-lapse simulation results for single stage 3-input majority gate

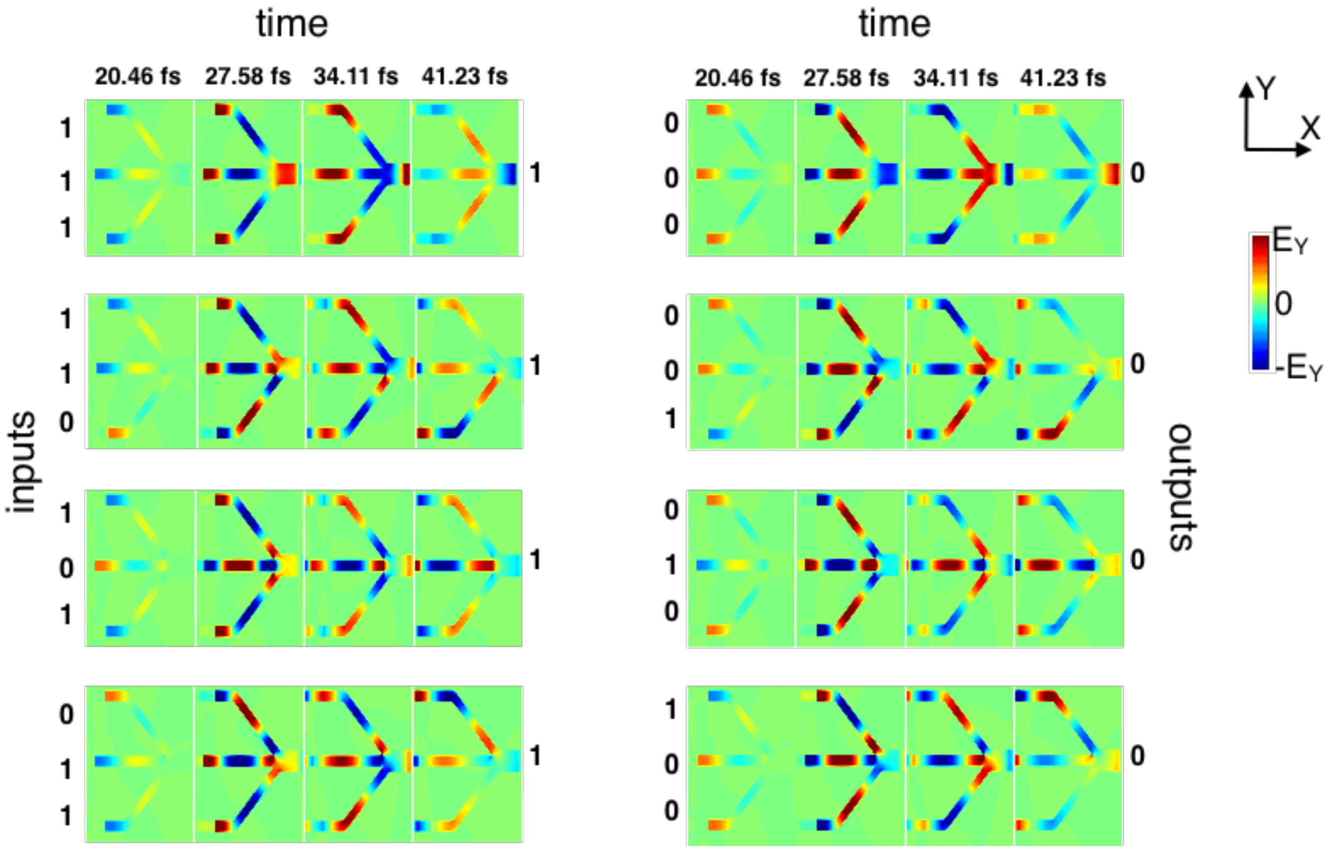

Fig. S3. Time-lapse simulation results in terms of the distribution of the electric field component E_Y in the x-y plane

S5. Dimensional scaling at each stage

We use the same layout for the 3-input majority gate as seen in Fig. 2(b) of the main paper at each stage but with appropriate dimensional scaling as shown in Fig. 4. Since the pitch follows a scaling of *p, 3p* and *9p* as seen in Fig. 4(a), to keep the same small merging angle of 35° between the waveguides, we also scale the length of the combiner region (x_2) accordingly. Note that due to the increase in the length of bends, the path difference ($\delta l_1, \delta l_2$ and δl_3) at each stage has to be considered separately and should be adjusted in the middle arm. For improved transmission via impedance matching, we choose the widths of the waveguides at each stage as *w* (=60 nm), 2*w* and 3*w* respectively. Note that we resort to a non-aggressive scaling of the widths since beyond 3*w* (=180 nm), the coupled MIM mode tends to split into two separate modes. However, we still gain from the fact that by increasing the widths, we increase the propagation length L_P of the SPP from 5.3 μm in the first stage to 8.46 μm in the second and 10.76 μm in the third. This proves to be beneficial since the overall size of the majority gate increases from one stage to the next.

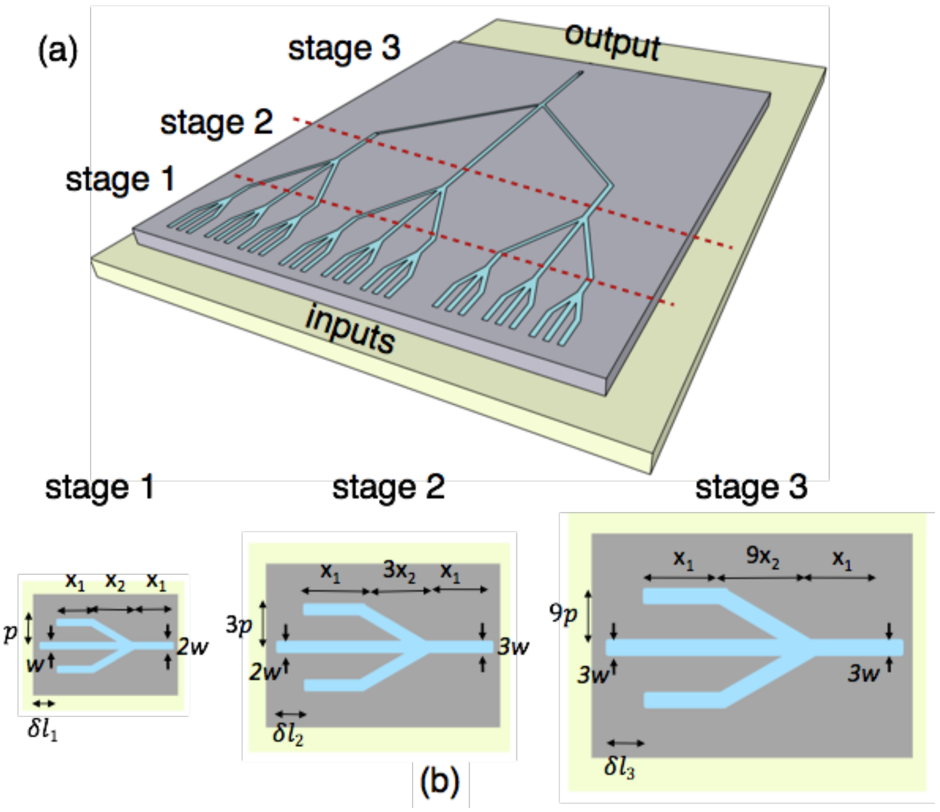

Figure 4. (a, b) Dimensional scaling and layout for cascaded majority gate structure.

S6. Time-Lapse Simulation Results for 2-Stage Cascaded Majority Logic Gate

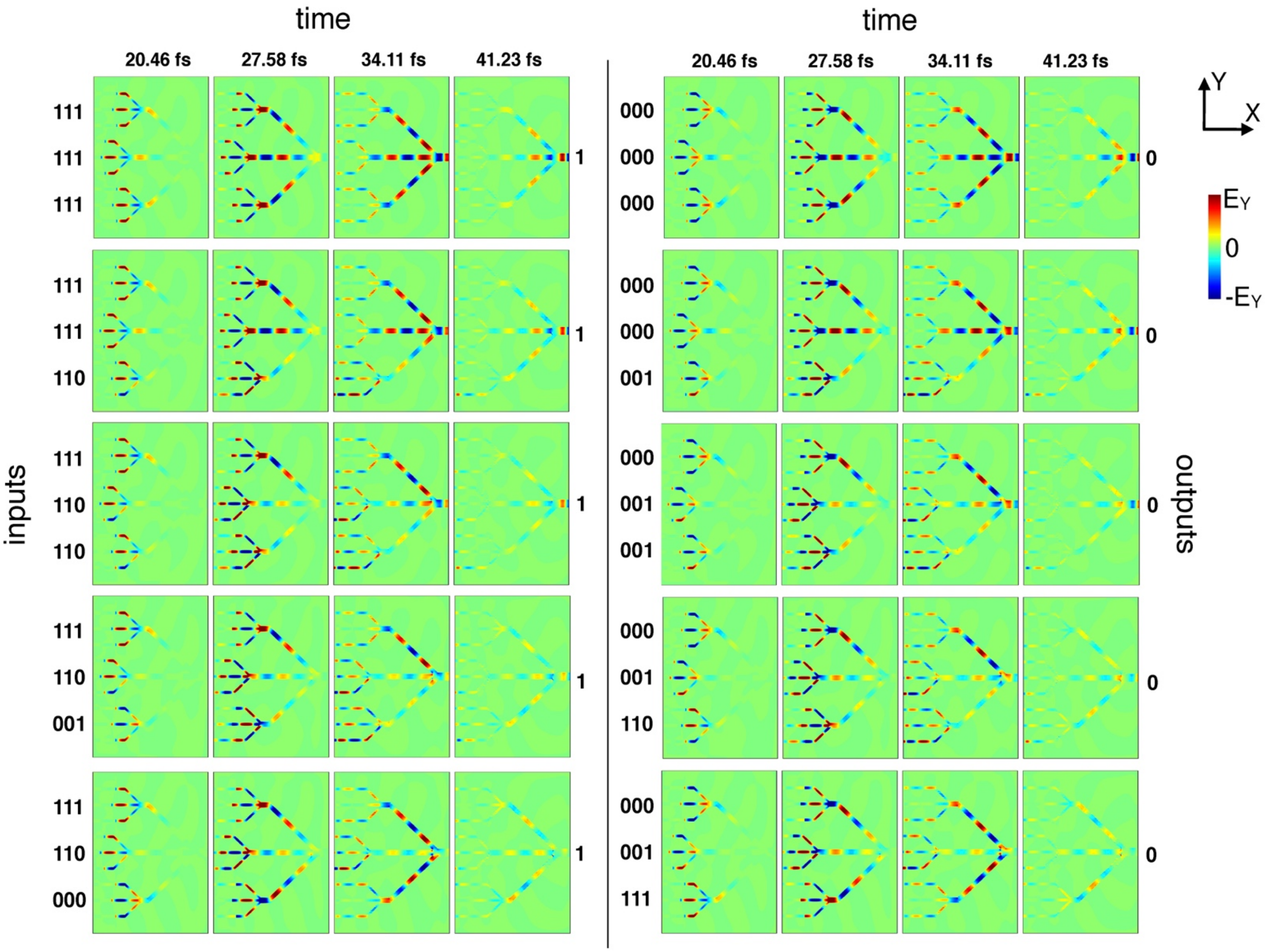

Figure S5. Time-lapse simulation results in terms of the distribution of the electric field component E_Y in the x-y plane

S7. Time-Lapse Simulation Results for 2-Stage Cascaded Majority Logic Gate with Reference

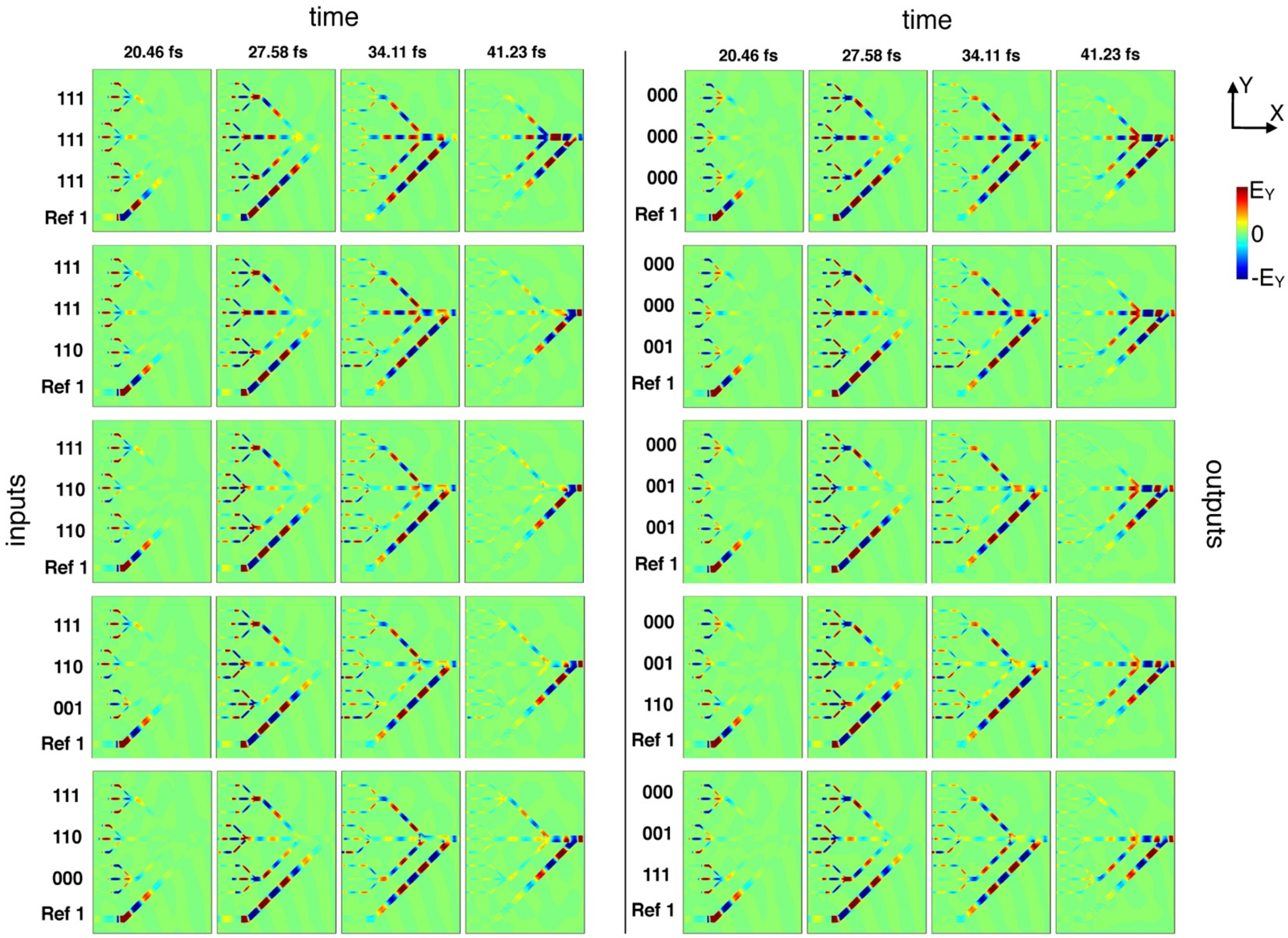

Figure S6. Time-lapse simulation results in terms of the distribution of the electric field component E_Y in the x-y plane

S8. Simulation results for single stage 3-input majority gate with narrowband excitation

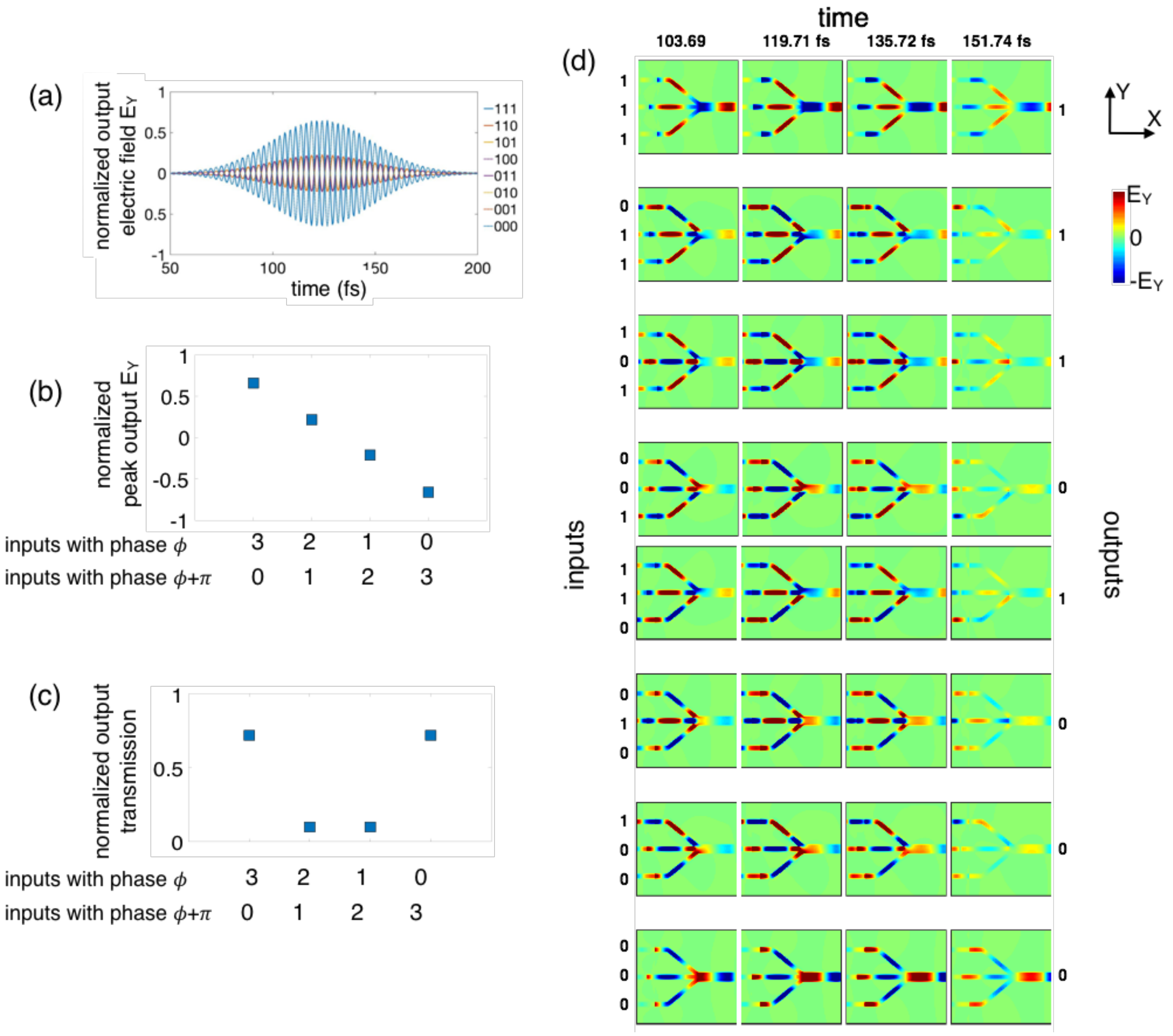

Figure S7. (a) Simulation result for a 3-input plasmonic majority gate for 2^3 input combinations in terms of the time-domain electric field component E_Y at the output, normalized to the total source electric field and averaged over the cross-section of the output waveguide. A mode source with narrowband excitation is used as mentioned in section S2. (b, c) Calculated peak values of the normalized average electric field component E_Y and normalized transmitted power at the output for different combinations of the input phases. (d) Time-lapse simulation results in terms of the distribution of E_Y in the x-y plane showing the propagation and interference of the SPP waves.

References

1 Ebbesen, T. W., Genet, C. & Bozhevolnyi, S. I. Surface-plasmon circuitry. *Physics Today* (2008).

2 Maksymov, I. S. & Kivshar, Y. S. Broadband light coupling to dielectric slot waveguides with tapered plasmonic nanoantennas. *Optics letters* **38**, 4853-4856 (2013).

3 Panchenko, E., James, T. D. & Roberts, A. Modified stripe waveguide design for plasmonic input port structures. *Journal of Nanophotonics* **10**, 016019-016019 (2016).

4 Koller, D. *et al.* Organic plasmon-emitting diode. *Nature Photonics* **2**, 684-687 (2008).

5 Neutens, P., Lagae, L., Borghs, G. & Van Dorpe, P. Electrical excitation of confined surface plasmon polaritons in metallic slot waveguides. *Nano letters* **10**, 1429-1432 (2010).

6 Walters, R. J., van Loon, R. V., Brunets, I., Schmitz, J. & Polman, A. A silicon-based electrical source of surface plasmon polaritons. *Nature Materials* **9**, 21-25 (2010).

7 Cazier, N. *et al.* Electrical excitation of waveguided surface plasmons by a light-emitting tunneling optical gap antenna. *Optics express* **24**, 3873-3884 (2016).

8 Uskov, A. V., Khurgin, J. B., Protsenko, I. E., Smetanin, I. V. & Bouhelier, A. Excitation of plasmonic nanoantennas by nonresonant and resonant electron tunnelling. *Nanoscale* **8**, 14573-14579 (2016).

9 Kern, J. *et al.* Electrically driven optical antennas. *Nature Photonics* **9**, 582-586 (2015).

10 Papaioannou, S. *et al.* Active plasmonics in WDM traffic switching applications. *Scientific reports* **2**, 652 (2012).

11 Im, S.-J. *et al.* Plasmonic phase modulator based on novel loss-overcompensated coupling between nanoresonator and waveguide. *Scientific reports* **6** (2016).

12 Lumerical, F. Solutions. *Web source [https://www/. lumerical. com/tcad-products/fdtd/]* (2012).

13 Cai, W., Shin, W., Fan, S. & Brongersma, M. L. Elements for Plasmonic Nanocircuits with Three-Dimensional Slot Waveguides. *Advanced materials* **22**, 5120-5124 (2010).

14 Veronis, G. & Fan, S. Bends and splitters in metal-dielectric-metal subwavelength plasmonic waveguides. *Applied Physics Letters* **87**, 131102 (2005).

15 Kocabas, S. E., Veronis, G., Miller, D. A. & Fan, S. Transmission line and equivalent circuit models for plasmonic waveguide components. *IEEE Journal of Selected Topics in Quantum Electronics* **14**, 1462-1472 (2008).
